# Relation between tag position and degree of visualized cerebrospinal fluid reflux into the lateral ventricles in time-spatial labeling inversion pulse magnetic resonance imaging at the foramen of Monro

**DOI:** 10.1186/s12987-015-0011-0

**Published:** 2015-06-21

**Authors:** Erik H Middlebrooks, Jeffrey A Bennett, Alissa Old Crow

**Affiliations:** Department of Radiology, College of Medicine, University of Florida, 1600 SW Archer Rd., PO Box 100374, Gainesville, FL 32610 USA; K. Scott and E.R. Andrew Advanced Neuroimaging Lab, University of Florida, Gainesville, FL USA

**Keywords:** Magnetic resonance imaging, Cerebrospinal fluid, Physiology, Spin labels

## Abstract

**Background:**

Time-spatial labeling inversion pulse (Time-SLIP) magnetic resonance imaging allows non-invasive visualization of cerebrospinal fluid (CSF) movement. Our study evaluated the sensitivity of the Time-SLIP tag placement on the measurement of CSF reflux from the third ventricle into the lateral ventricles via the foramen of Monro.

**Findings:**

Multiple Time-SLIP MRI scans were obtained in three healthy volunteers (23–55 years of age) evaluating the observed CSF pulsation and reflux from the third ventricle into the lateral ventricles while varying the placement of the tag. Linear regression was performed to evaluate the effects of tag position on the amount of visualized reflux and pulsation. Variation in the position of the tag relative to the plane of the free margin of the septum pellucidum produced a significant inverse variation in the observed reflux into the lateral ventricles (R^2^ = 0.74). The further the distance of the top (superior edge) of the tag from the plane of the free margin of the septum pellucidum, the less reflux into the lateral ventricles was observed (*P* = 0.006).

**Conclusions:**

The amount of observed CSF reflux into the lateral ventricles in Time-SLIP MR imaging is dependent on the positioning of the CSF tag with decreasing amount of visualized reflux the further caudal the CSF tag is relative to the free margin of the septum pellucidum.

**Electronic supplementary material:**

The online version of this article (doi:10.1186/s12987-015-0011-0) contains supplementary material, which is available to authorized users.

## Findings

### Background

Cerebrospinal fluid (CSF) plays a pivotal role in normal physiology of the human central nervous system (CNS). CSF is thought to perform many functions such as supplying mechanical support for the brain, contributing to maintenance of intracranial pressure, stability against traumatic forces, and in clearance of metabolic by-products [1, 2]. With an understanding of the normal physiologic role of CSF, it is apparent that abnormalities in CSF function may serve as a basis for underlying diseases of the brain. Previous research has demonstrated impaired CSF flow in many diseases including, but not limited to, normal pressure hydrocephalus (NPH) [3], Alzheimer’s disease [4], Chiari malformation [5], and multiple sclerosis [6].

MR sequences such as phase-contrast imaging (PC-MRI) have had a substantial impact on our ability to monitor changes in CSF flow [7, 8]. Yamada et al. [8] recently repurposed an arterial spin labeling variant known as time-spatial labeling inversion pulse (Time-SLIP) to noninvasively visualize CSF flow without relying on velocity-dependent phase changes as in PC-MRI. Time-SLIP utilizes a nonselective inversion recovery pulse to invert all longitudinal magnetization in the field-of-view followed by a second spatially-selective inversion pulse (tag pulse). This spatially-selective pulse results in equilibrium magnetization of only the fluid and tissue located within the user-selected tag region. Immediately following the selective tag pulse, the magnetization outside of the tag will begin to relax back towards equilibrium. As the background magnetization approaches the null point, images can be acquired using a single-shot fast spin-echo sequence. Magnetization that originated within the user-selected tag will appear bright whereas magnetization outside of the tag will appear dark. CSF that has traveled outside of the tag during the recovery time will be visualized easily against the dark signal of tissue that did not originate within the tag. Repeated images are then obtained at different recovery times, triggered by cardiac gating with a photoplethysmogram, allowing visualization of CSF displacement [9–11].

The purpose of our study is to evaluate the effects of tag placement on the measurement of CSF reflux from the third ventricle into the lateral ventricles via the foramen of Monro. This normal physiologic phenomenon has been reported to be abnormal in various clinical conditions such as normal pressure hydrocephalus and noncommunicating hydrocephalus [9, 10]. The results of the study would ideally determine if the tag placement adversely affects reflux measurements and if an optimal position for tag placement exists. This may avert erroneous interpretation of CSF reflux changes due to incorrect tag placement.

## Methods

### Subjects

Our HIPAA-compliant study met criteria for exemption from full review of the University Institutional Review Board. Informed consent was waived for retrospective review of pre-existing data. Three healthy volunteers ranging from 23 to 55 years of age were imaged.

### Image acquisition

Imaging was performed on a Vantage Titan 3T MRI (Toshiba, Tokyo, Japan) with a 32-channel head coil. The Time-SLIP sequence was obtained in an oblique coronal plane oriented parallel to the long-axis of the foramen of Monro (See Additional file 1: Figure S1 MR images showing the selection of imaging plane). The oblique coronal plane was determined by initially obtaining a 3D sagittal localizer to identify the foramen of Monro. The localizer was a 3D fast advanced spin echo (FASE) with a TR of 6,000 ms and TE of 153 ms. The oblique coronal plane was then selected as the plane through the foramen of Monro which is parallel to the long axis of the foramen.

### Sequence parameters

The Time-SLIP sequence utilized a TR of approximately 13,000 ms (15 R–R intervals to allow for maximum CSF recovery) and a TE of 80 ms. The matrix size was 224 × 224 with a field of view of 23 × 23 cm and slice thickness of 5-mm. Electrocardiographic time delay from an R wave to the initiation of the nonselective inversion recovery pulse was 0 ms. The subsequent 2-D single-shot FSE images were obtained within one acquisition with variable TIs increasing in increments of 200 ms for a total of 15 times starting at 2,000 ms. The acquisition time for each individual run averaged about 3.5 min depending on pulse rate.

### Measurements

Multiple scans were obtained in each subject with the tag varying in position. The top (superior) of the tag was placed in locations ranging from 8.5 mm below the free margin of the septum pellucidum (FMSP) to 13 mm above the FMSP. Images were reviewed at a dedicated radiology imaging workstation. The distance from the top (superior aspect) of the tag and the plane of the FMSP was determined. The plane of the FMSP was defined as a perpendicular line tangential to the inferior-most aspect of the septum pellucidum (see Additional file 2: Figure S2. MR images showing the plane of FMSP and normal reflux). For each acquisition, two additional measurements were made. The first measurement was the greatest cephalad height that the CSF pulsated above the plane of the tagged fluid across all time points. The second measurement was the amount of CSF reflux into the lateral ventricles determined by the height of CSF reflux above the plane of the FMSP (see Additional file 3: Figure S3. Measurement of CSF pulsation.).

### Statistical analysis

Statistical analysis was performed with Prism 6.0 (GraphPad Software, Inc. San Diego, CA, USA). Data was analyzed with a linear regression model to determine correlations between measurements of CSF displacement relative to the placement of the CSF tag. Only those measurements with the tag placed below the plane of the FMSP were used in regression analysis of the reflux height above the plane of the FMSP.

## Results

**Figure 1:**
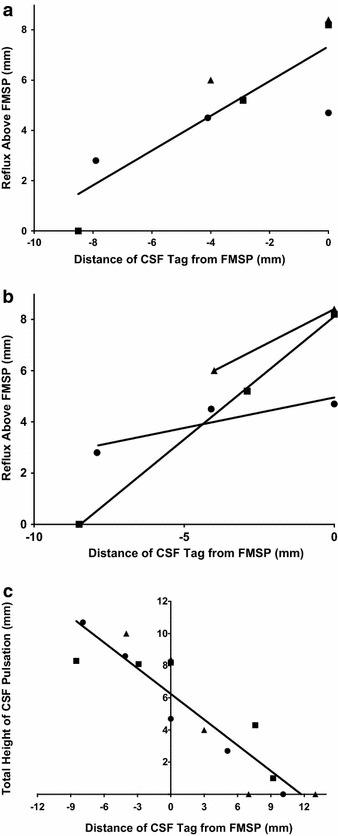
**a** Effect of CSF tag position on the amount of visualized CSF reflux from the 3rd ventricle into the lateral ventricles. Measurements are relative to the plane of the free margin of the septum pellucidum (FMSP). As the tag position is situated closer to the FMSP, there is an increase in visualized CSF reflux (*P* = 0.006). **b** The effect of CSF tag position on the amount of visualized CSF reflux on an individual subject level. The same effect of increasing reflux as the tag is moved closer to the FMSP is illustrated in each individual subject. **c** Effect of CSF tag position on the height of visualized CSF pulsation above the tag. Higher CSF pulsation is seen as the tag position is moved caudally (*P* < 0.0001). *Triangle* subject 1, *square* subject 2, *circle* subject 3.

Variation in the position of the tag relative to the plane of the FMSP produced a significant variation in the measured reflux into the lateral ventricles (Figure 1a). The amount of reflux above the plane of the FMSP was inversely correlated with distance of the tag placement from the plane (y = 0.69x + 7.3; R^2^ = 0.74). That is, the further the distance of the top (superior edge) of the tag from the plane of the FMSP, the less visualized reflux into the lateral ventricles (*P* = 0.006). Analysis of each individual subject also confirmed a progressive decrease in visualized reflux with increasing distance from the FMSP within each subject (Figure 1b). We found no significant measurable difference between maximum reflux height observed between the right and left sides of the brain across all measurements.

The position of the tag also had a measurable effect on the visualized total height of CSF pulsation (Figure 1c). As the tag was moved caudally from a position originating in the upper aspect of the lateral ventricles, the total height of CSF pulsation above the tag increased (y = 0.53x + 6.2; R^2^ = 0.84; *P* < 0.0001).

**Figure 2:**
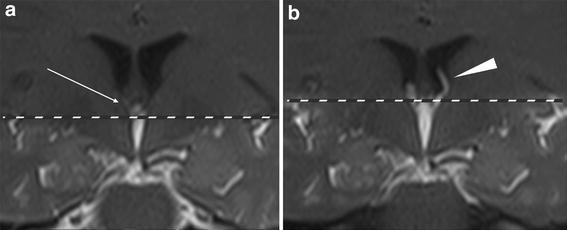
Coronal Time-SLIP MR images in the same subject illustrating the effect of improper CSF tag placement. **a** When the *top* of the tag (*dashed line*) was placed more inferior to the free margin of the septum pellucidum (FMSP), minimal CSF reflux (*arrow*) was seen into the lateral ventricles. **b** Once the *top* of the tag (*dashed line*) was moved to the plane of the FMSP, reflux was easily identified into the lateral ventricles (*arrowhead*).

Despite increasing height of pulsation as the tag is moved caudally, there continued to be a decrease in the amount of observed reflux into the lateral ventricles (Figure 2). We identified reflux in some instances with the tag placed well below the FMSP. However, it is evident that the reflux was underestimated as each subject had decreasing reflux as the distance from the FMSP increased (Figure 1b).

## Discussion

The results of our study show a substantial variation in the amount of observed reflux from the third ventricle into the lateral ventricles based upon the position of the CSF tag in Time-SLIP imaging. For demonstration of reflux into the lateral ventricles, we found that the optimum tag location is with the top (superior) border of the tag at the level of the FMSP.

We postulate the explanation of these findings is that the majority of CSF exchanged between the third and lateral ventricles during normal pulsation is from fluid located in the cephalad portion of the third ventricle. Fluid tagged in the more caudal portion of the third ventricle contributes less to the overall exchanged pool of fluid. An alternative hypothesis would be that fluid closer to the foramen experiences a higher reflux velocity resulting in an apparent increase in reflux height.

Modern investigation into the normal flow of CSF has suggested a much more complex circulation than was historically thought [12–15]. In vivo imaging techniques have promoted the idea of a pulsatile nature of CSF flow as opposed to the classic theory of bulk flow from the choroid plexus of the ventricular system outward into the subarachnoid space [13, 16]. The pulsatile nature results in bidirectional flow through the ventricular system which can drive fluid from the subarachnoid space back as far as the lateral ventricles [17]. This system drives the regulation of intracranial pressure and the clearance of metabolites, many of which are pro-inflammatory or toxic to the brain substance [1, 2]. Since the brain lacks true lymphatic vasculature, the majority of interstitial fluid clearance is through the CSF [1, 2].

Disturbance of the normal pulsatile nature of CSF flow has been shown to correlate with many diseases of the CNS. Recent studies have confirmed decreased reflux from the third ventricle into the lateral ventricles in conditions such as aqueductal stenosis [18], noncommunicating hydrocephalus [9], and normal pressure hydrocephalus [11]. It is also possible that alterations in normal CSF physiology play a role in the pathogenesis of other diseases of the CNS. The application of techniques described in this study may prove useful in further elucidating both the normal physiologic flow of CSF, as well as in assessment of underlying pathologic disturbances.

The advent of MRI sequences sensitive to flow effects has greatly advanced our understanding of normal and abnormal CSF flow patterns. One of the most commonly used MRI pulse sequences is phase-contrast imaging (PC-MRI) [7, 8]. Phase-contrast MRI relies on the detection of complex phase changes in moving fluid [7]. An advantage of PC-MRI is the ability to quantify CSF flow [7, 11]. A primary disadvantage of PC-MRI, however, is that turbulent CSF flow [11, 19, 20] and bulk CSF flow [11, 21] cannot be reliably visualized. Time-SLIP imaging allows the noninvasive visualization of CSF displacement without relying on velocity-dependent phase changes. The principle underlying time-SLIP imaging is visualization of fluid displacement by selective magnetic tagging of protons. This technique can be applied to visualize the amount of fluid displacement from the third ventricle into the lateral ventricles. The bidirectional flow through the foramen of Monro related to the cardiac and respiratory cycle results in the mixing of tagged protons in the third ventricular CSF refluxing back into the lateral ventricles. This technique allows improved characterization of turbulent flow and CSF stasis relative to phase-contrast techniques.

We have shown that the amount of visualized CSF reflux into the lateral ventricles in Time-SLIP imaging is dependent on the positioning of the CSF tag. To the authors’ knowledge, there are no existing publications describing the distance dependence of Time-SLIP tag placement on the amount of visualized CSF reflux. Our results suggest caution be exercised when interpreting a lack of reflux as pathologic when the tag position is not optimal. We have also illustrated that inappropriate tag positioning may continue to show CSF reflux, but the amount of reflux will be underestimated. The information presented herein is essential for future studies assessing the physiologic and pathologic variability of this phenomenon.

## Conclusion

The amount of observed CSF reflux into the lateral ventricles in Time-SLIP imaging is dependent on the positioning of the CSF tag with decreasing amount of reflux the further caudal the CSF tag is relative to the free margin of the septum pellucidum.

## Additional files

Additional file 1:
**Figure S1.** MR images showing the selection of imaging plane. (A) Sagittal localizer illustrates the location and orientation of the oblique coronal imaging plane (line) which is parallel to the foramen of Monro (arrowhead). (B) Coronal localizer demonstrating the ideal plane for illustration of reflux of CSF from the third ventricle into the lateral ventricles. The coronal oblique plane is chosen to visualize the foramen of Monro (arrow). Additional file 2:
**Figure S2.** (A) MR images showing the plane of the free margin of the septum pellucidum (line) perpendicular to the free margin of the septum pellucidum (arrow). (B) Normal reflux into the lateral ventricle is demonstrated (arrow). Additional file 3:
**Figure S3.** Measurement of CSF pulsation. Example of recorded measurements including measurement of the total height of CSF pulsation (larger double-headed arrow) above the CSF tag (dashed line) and height of CSF reflux into lateral ventricle above the plane of the free margin of the septum pellucidum (small double-headed arrow).

## Abbreviations

Time-SLIP: time-spatial labeling inversion pulse
CSF: cerebrospinal fluid
PC-MRI: phase-contrast magnetic resonance imaging
FMSP: free margin of the septum pellucidum
