# Supplementary figures and images for: Relation between tag position and degree of visualized cerebrospinal fluid reflux into the lateral ventricles in time-spatial labeling inversion pulse magnetic resonance imaging at the foramen of Monro
Source: Fluids Barriers CNS. 2015 Jun 21;12:14. doi: 10.1186/s12987-015-0011-0 (PMC4475619; doi:10.1186/s12987-015-0011-0)

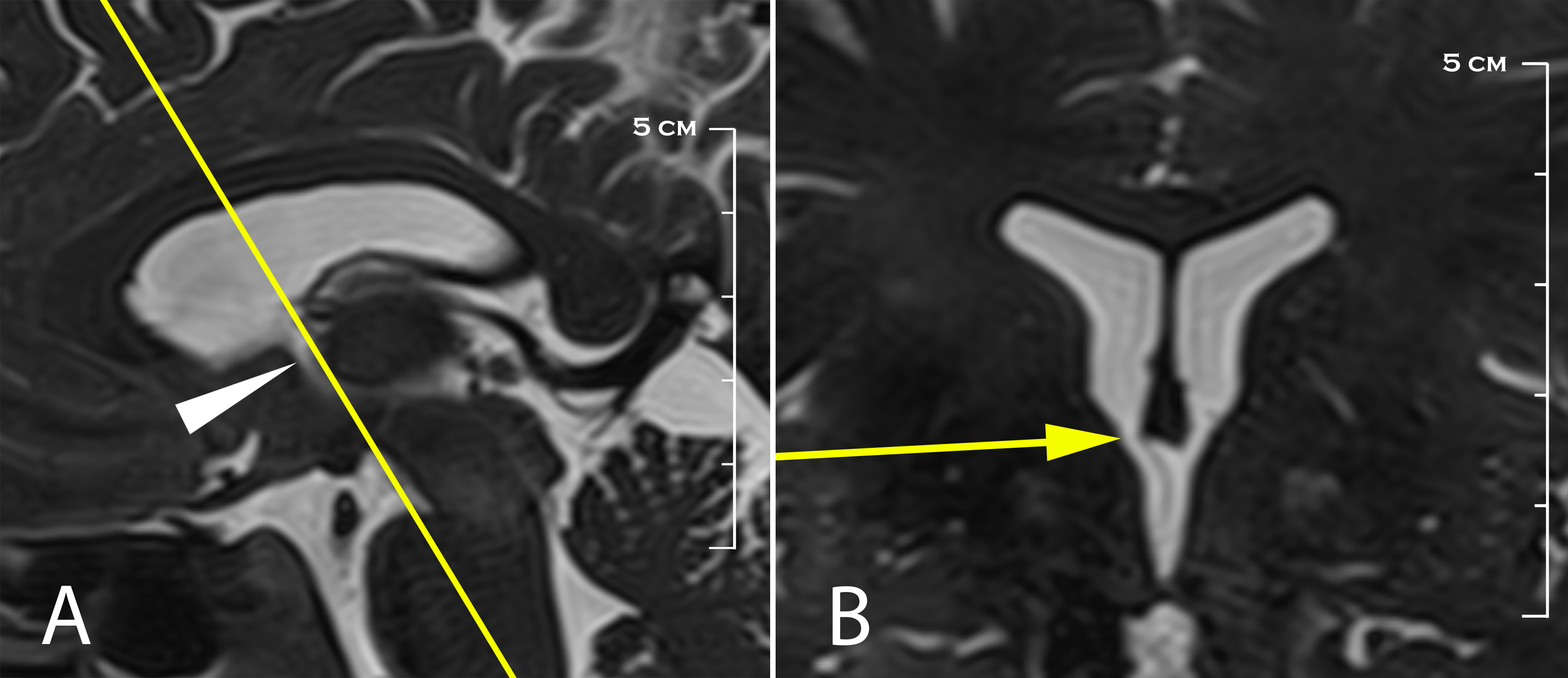

Supplement: Additional file 1: — Figure S1. MR images showing the selection of imaging plane. (A) Sagittal localizer illustrates the location and orientation of the oblique coronal imaging plane (line) which is parallel to the foramen of Monro (arrowhead). (B) Coronal localizer demonstrating the ideal plane for illustration of reflux of CSF from the third ventricle into the lateral ventricles. The coronal oblique plane is chosen to visualize the foramen of Monro (arrow). [file 12987_2015_11_MOESM1_ESM.tiff]

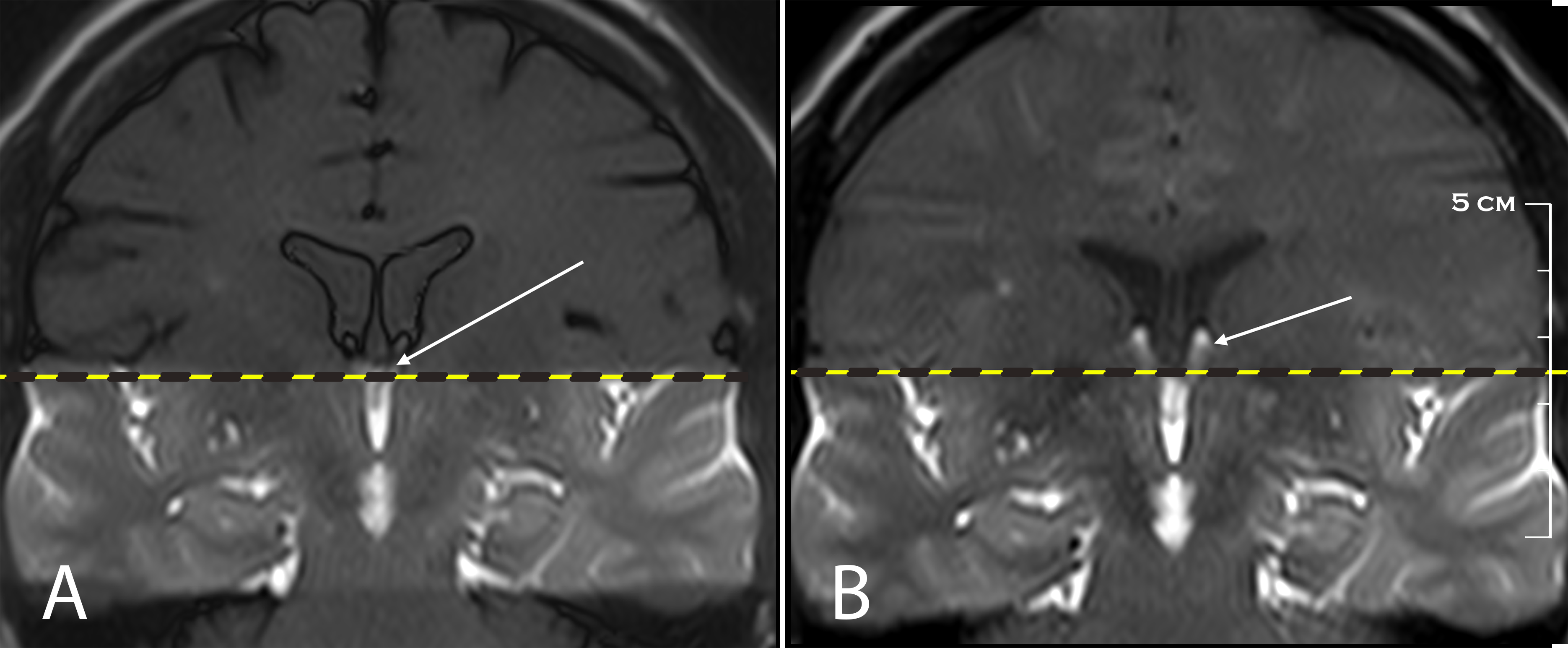

Supplement: Additional file 2: — Figure S2. (A) MR images showing the plane of the free margin of the septum pellucidum (line) perpendicular to the free margin of the septum pellucidum (arrow). (B) Normal reflux into the lateral ventricle is demonstrated (arrow). [file 12987_2015_11_MOESM2_ESM.tiff]

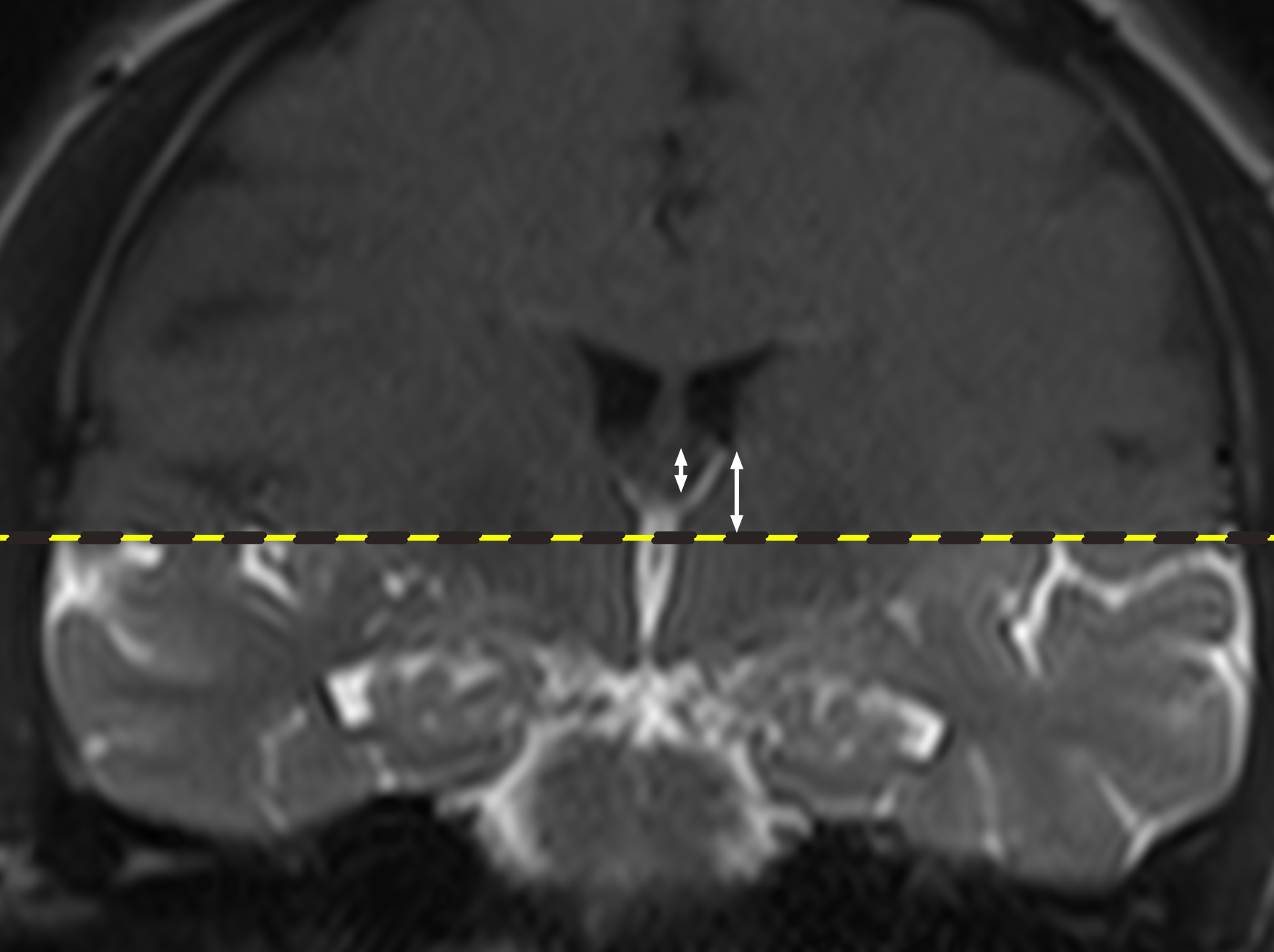

Supplement: Additional file 3: — Figure S3. Measurement of CSF pulsation. Example of recorded measurements including measurement of the total height of CSF pulsation (larger double-headed arrow) above the CSF tag (dashed line) and height of CSF reflux into lateral ventricle above the plane of the free margin of the septum pellucidum (small double-headed arrow). [file 12987_2015_11_MOESM3_ESM.tiff]
